# Supplementary material for: Environmental signals rather than layered ontogeny imprint the function of type 2 conventional dendritic cells in young and adult mice
Source: Nat Commun. 2021 Jan 19;12:464. doi: 10.1038/s41467-020-20659-2 (PMC7815729; doi:10.1038/s41467-020-20659-2)
Supplement: Supplementary file 1 — Supplementary Information [file 41467_2020_20659_MOESM1_ESM.pdf]

Supplementary information

Environmental signals rather than layered ontogeny imprint the function of type 2 conventional dendritic cells in young and adult mice

First author: Nikos E. Papaioannou
Corresponding author: Barbara U. Schraml

Supplementary Figure 1

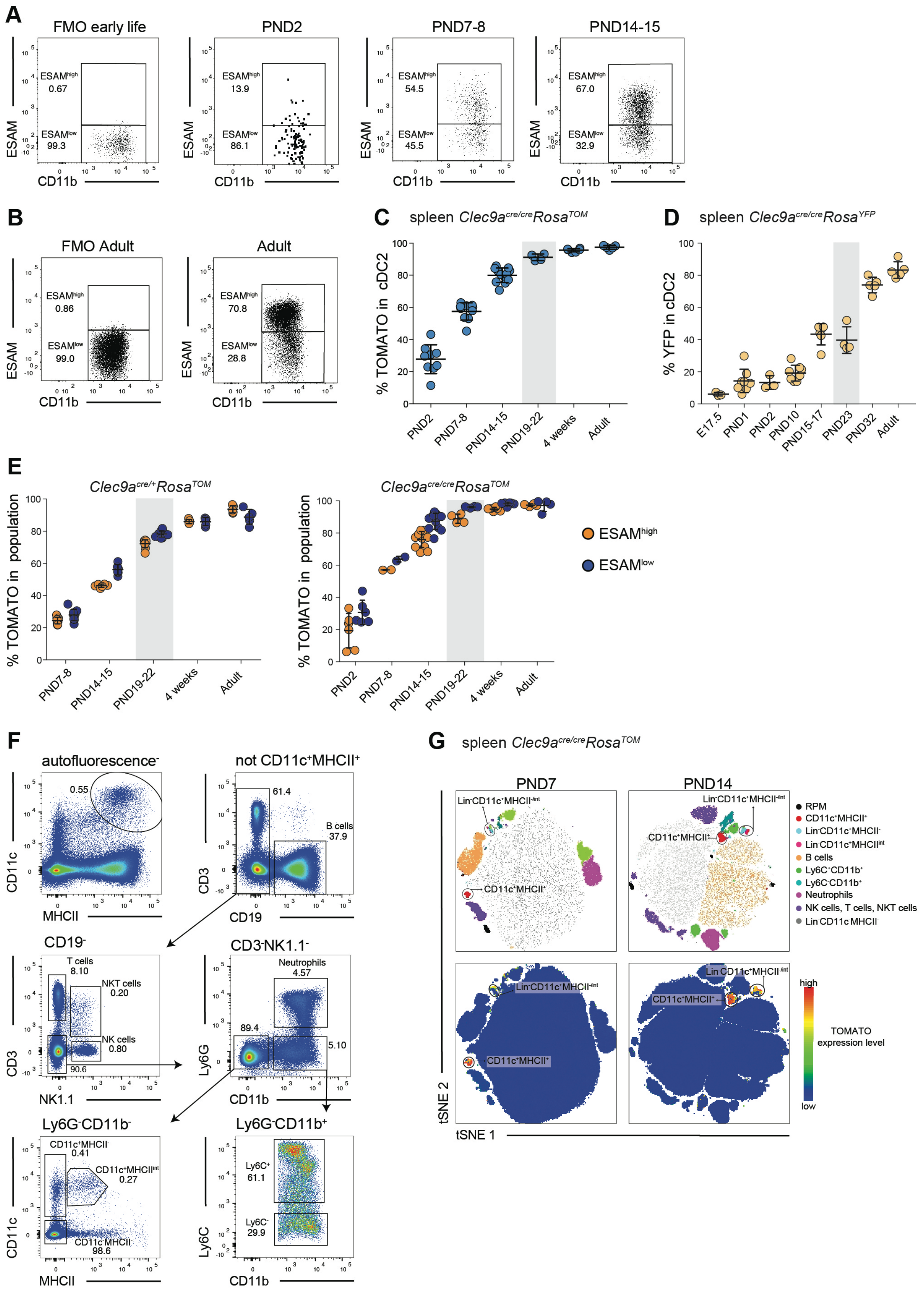

Supplementary Figure 1. ***Clec9^{cre}* mice faithfully trace cDCs in early life. (A, B**) Splenic cDC2 (CD11c^{+}MHCII^{+}CD11b^{+}) from *Clec9a$^{cre/+}$Rosa^{TOM}* or *Clec9a$^{cre/cre}$Rosa^{TOM}* mice of the indicated ages were analyzed for ESAM expression. Fluorescence minus one (FMO) controls used to set the gates are shown. **(C-E)** Splenic cDC2 from *Clec9a$^{cre/cre}$Rosa^{TOM}* **(C)** and *Clec9a$^{cre/cre}$Rosa^{YFP}* mice **(D)** were analyzed for TOMATO and YFP expression at the indicated ages (n=9, PND2; n=10, PND7-8; n=12, PND14-15; n=4, PND19-22; n=6, 4 weeks; n=5, adult **(C)** and n=3, E17.5; n=8, PND1; n=3, PND2; n=8, PND10; n=6, PND15-17; n=4, PND23; n=5, PND32; n=5, adult **(D)**). **(E)** Splenic ESAM^{high} and ESAM^{low} cDC2 from *Clec9a$^{cre/+}$Rosa^{TOM}* (left) and *Clec9a$^{cre/cre}$Rosa^{TOM}* (right) mice were analyzed for TOMATO expression at the indicated ages (n=9, PND7-8; n=6, PND14-15; n=10, PND19-22; n=5, 4 weeks; n=5, adult for left plot and n=6, PND2; n=2, PND7-8; n=12, PND14-15; n=4, PND19-22; n=6, 4 weeks; n=4, adult for right plot). Each dot represents one mouse, horizontal bars represent mean, error bars represent SD, grey rectangles indicate weaning. **(F, G)** Spleens from one- and two-week-old *Clec9a$^{cre/cre}$Rosa^{TOM}* mice were analyzed by flow cytometry as indicated, to profile TOMATO expression in lymphoid and myeloid populations. **(F)** Representative gating strategy. **(G)** t-Distributed Stochastic Neighbor Embedding (tSNE) of splenocytes from PND7 and PND14 *Clec9a$^{cre/cre}$Rosa^{TOM}* mice. Cells were clustered independent of TOMATO. Manually gated populations were overlaid on the tSNE plot and indicated by color. Blue-to-red gradient indicates increasing intensity of TOMATO expression. Source data are provided as a Source Data file.

Supplementary Figure 2

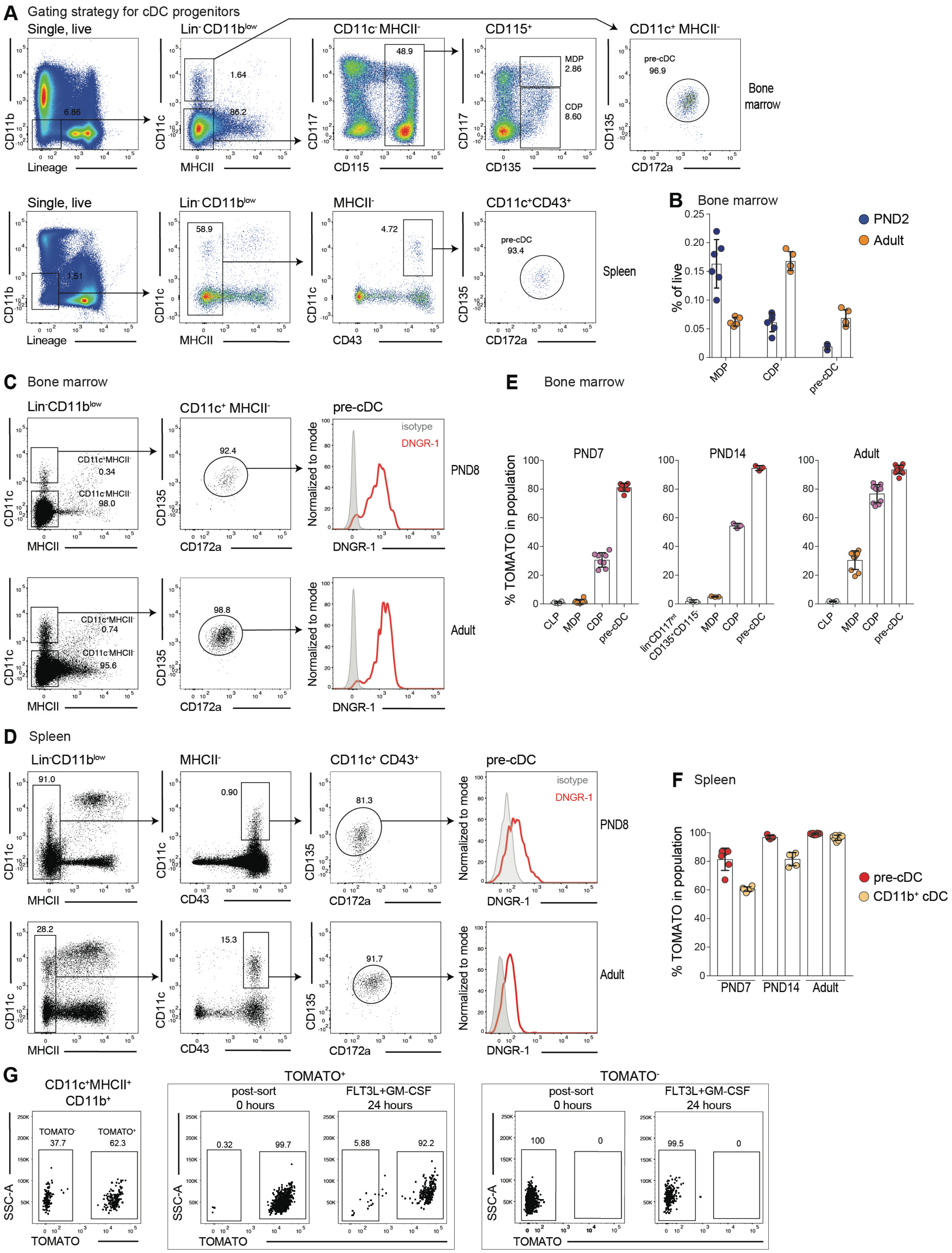

Supplementary Figure 2. Identification of cDC progenitors in early life. (A) Representative gating strategy for the identification of cDC progenitors in bone marrow (upper row) and spleen (lower row) of adult mice. **(B)** Frequency of cDC progenitors as identified in **(A)** in the bone marrow of wild type mice at the indicated ages (n=6, MDP and CDP PND2; n=3, pre-cDC PND2; n=5, adult). Each dot represents one biological replicate, horizontal bars represent mean, error bars represent SD. **(C, D)** Bone marrow **(C)** and spleen **(D)** from wild type mice of the indicated ages were analyzed by flow cytometry. Lin$^-$ cells were gated, pre-cDCs were identified as indicated and stained with anti-DNGR-1 (red) or isotype-matched control antibody (grey). Lineage: CD3, B220, NK1.1, CD4, CD8α, TER119. **(E, F)** Bone marrow **(E)** and spleen **(F)** of *Clec9a*$^{cre/cre}$*Rosa*^{TOM} mice was analyzed by flow cytometry at the indicated ages. **(E)** CLPs (lin$^-$CD11b$^-$CD115$^-$ CD117^{int}Sca-1^{int}CD135$^+$CD127$^+$), MDPs (lin$^-$CD11c$^-$MHCII$^-$CD11b^{low} CD115$^+$ CD135$^+$ CD117^{high}), CDPs (lin$^-$CD11c$^-$MHCII$^-$CD11b^{low}CD115$^+$CD135$^+$CD117^{low}) and pre-cDCs (lin$^-$ MHCII$^-$CD11b^{low}CD11c$^+$CD135$^+$CD172a^{int}) were analyzed for TOMATO expression. The lin$^-$ CD11b$^-$CD117^{int}CD115$^-$CD135$^+$ fraction of bone marrows that contains CLPs was used to assess TOMATO expression in PND14 and adult mice (n=8, PND7; n=3, PND14; n=10, adult; n=4, CLP PND7 and adult). **(F)** TOMATO expression was analyzed in splenic pre-cDCs (lin$^-$MHCII$^-$ CD11b^{low}CD11c$^+$CD43$^+$CD135$^+$CD172a^{int}) and CD11b$^+$ cDCs (n=6, PND7; n=5, PND14; n=8, adult). Each dot represents one mouse, horizontal bars represent mean, error bars represent SD. Lineage: CD3, B220, NK1.1, CD4, CD8α, TER119. **(G)** TOM$^-$ and TOM$^+$ CD11b$^+$ cDC2 cells were sorted from spleens of one-week-old *Clec9a*$^{cre/cre}$*Rosa*^{TOM} mice and cultured *in vitro* with congenic CD45.1 filler splenocytes in the presence of FLT3L and GM-CSF. After 24 hours, TOM$^-$ and TOM$^+$ cells were analyzed for TOMATO labeling. FACS plots show TOMATO labeling in CD11b$^+$ cDC2 before sorting (left), sort purity of TOMATO$^-$ and TOMATO$^+$ fractions (right upper plots) and TOMATO labeling after 24 hours in culture (right bottom plots). Data are representative of two independent experiments with at least 2 biological replicates each. Source data are provided as a Source Data file.

Supplementary Figure 3

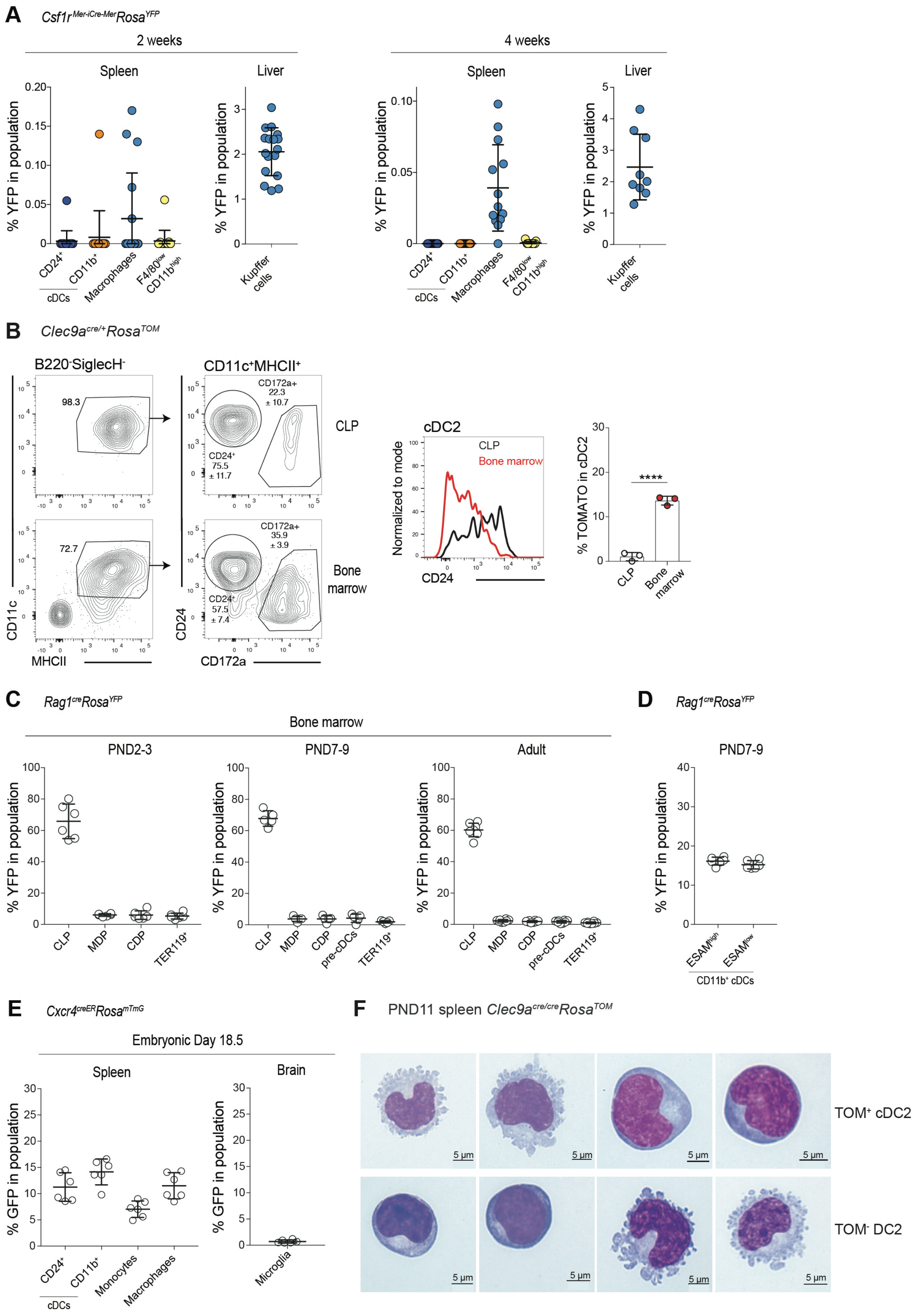

Supplementary Figure 3. Fate mapping reveals a lymphoid contribution to cDC2 in early life and TOM^{-} DC2 morphologically resemble TOM^{+} cDC2. (A) *Csf1r$^{Mer\text{-}iCre\text{-}Mer}$* dams were mated with male *Rosa^{YFP}* mice and injected with 4OH-tamoxifen on E8.5. Spleen and liver from offspring mice were analyzed by flow cytometry two (n=17) or four weeks (n=13, n=9 for liver) after birth. The percentage of YFP^{+} cells in the indicated populations is plotted. **(B)** Unfractionated bone marrow and 10^4 CLPs (lin^{-}CD11b^{-}CD115^{-}CD117^{int}CD135^{+}CD127^{+}B220^{-}TOMATO^{-}) from adult CD45.2^{+} *Clec9a$^{cre/+}$Rosa^{TOM}* mice were cultured with FLT3L for 7 days in the presence of CD45.1^{+} congenic bone marrow filler cells. Left: CD11c^{+}MHCII^{+} cells were identified by FACS and divided into CD24^{+} cDC1 and CD172a^{+} cDC2 equivalents. Numbers indicate mean frequency±SD. Middle: Expression of CD24 on cDC2 equivalents. Right: The frequency of TOMATO positive cells in cDC2 equivalents derived from bone marrow and CLP is shown. Data are representative of two independent experiments (n=3). Statistical analysis was performed using two-tailed t test. **(C, D)** Bone marrow **(C)** and spleen **(D)** from *Rag1^{cre}Rosa^{YFP}* mice at the indicated ages was analyzed by flow cytometry. **(C)** CLPs (lin^{-}CD11b^{-}CD115^{-}CD117^{int}Sca-1^{int}CD135^{+}CD127^{+}), MDPs (lin^{-}CD11c^{-}MHCII^{-}CD11b^{low}CD115^{+}CD135^{+}CD117^{high}), CDPs (lin^{-}CD11c^{-}MHCII^{-}CD11b^{low}CD115^{+}CD135^{+}CD117^{low}), pre-cDCs (lin^{-}MHCII^{-}CD11b^{low}CD11c^{+}CD135^{+}CD172a^{int}), as well as TER119^{+} erythroid cells were analyzed for YFP expression (n=6, PND2-3; n=5, PND7-9; n=8, adult; lineage: Ly6G, CD3, B220, NK1.1, CD8α). **(D)** cDC2 from spleen of *Rag1^{cre}Rosa^{YFP}* mice on PND7-9 were divided into ESAM^{high} and ESAM^{low} populations and analyzed for YFP expression (n=6). **(E)** Male *Cxcr4^{creER}* mice were mated with female *Rosa^{mTmG}* mice. Pregnant dams were injected with tamoxifen on E12.5. On E18.5 spleen and brain from offspring mice were analyzed by flow cytometry for GFP expression (n=6). Each dot in **(A-E)** represents one mouse, horizontal bars represent mean, error bars represent SD. **** $p<0.0001$. **(F)** Cytopsin analysis of splenic TOM^{+} cDC2 and TOM^{-} DC2 of 11-day-old *Clec9a$^{cre/cre}$Rosa^{TOM}* mice. Scale bar 5µm. Data are representative of two independent experiments. Source data are provided as a Source Data file.

Supplementary Figure 4

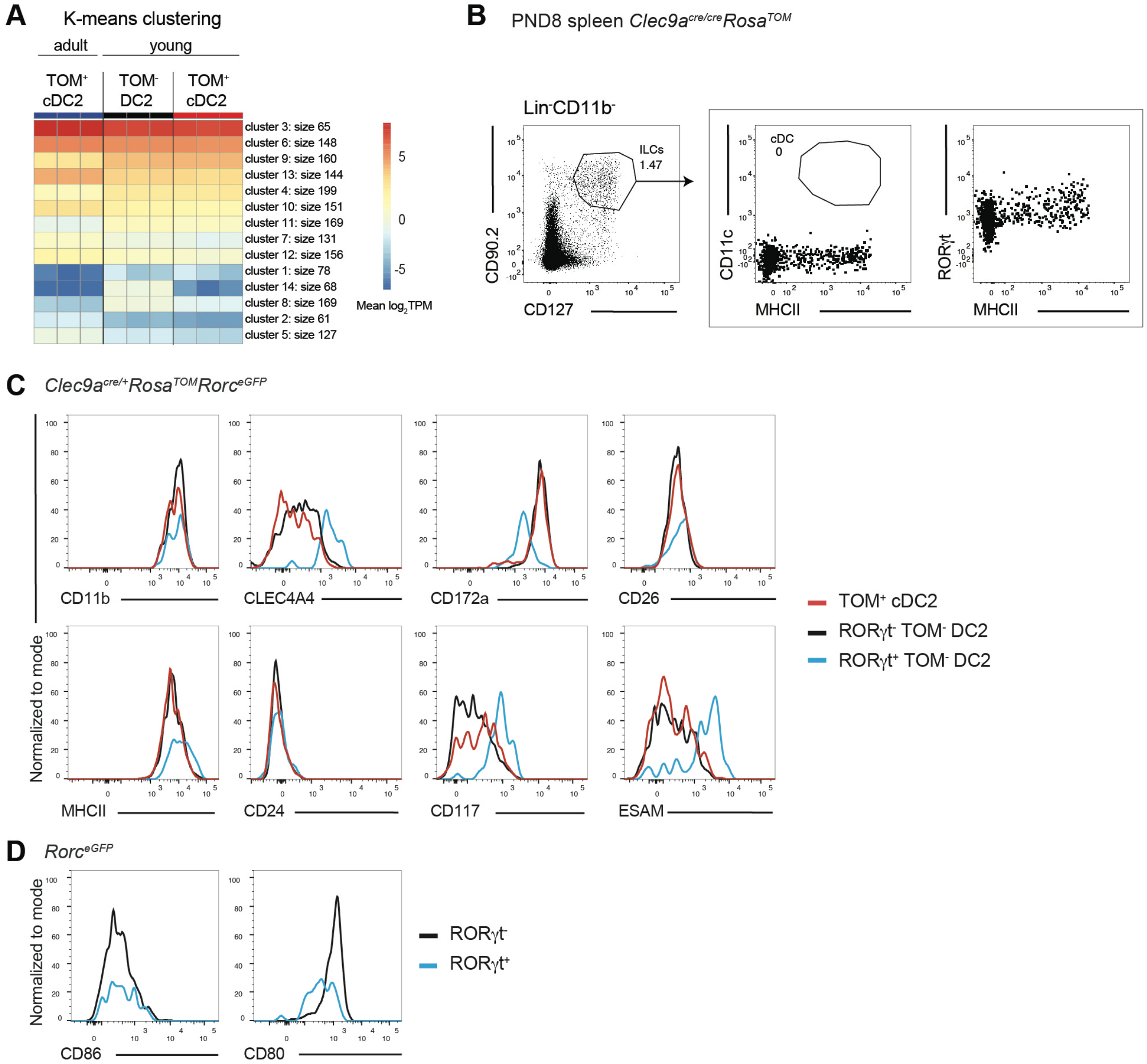

Supplementary Figure 4. RORγt$^+$TOM$^-$ DC2 in early life are distinct from ILCs and exhibit phenotypic profile that resembles ESAM^{hi} cDC2. **(A)** TOM$^-$ DC2 and TOM$^+$ cDC2 from 8-day-old and TOM$^+$ cDC2 from adult *Clec9a*$^{cre/cre}$*Rosa*^{TOM} mice were sorted and analyzed by mRNA sequencing. k-means clustering of differentially expressed genes (log_2 FC >1 and padj <0.05) between TOM$^+$ cDC2 from young mice, TOM$^-$ DC2 from young mice and TOM$^+$ cDC2 from adult mice. Significance of differentially expressed genes was tested using default commands of DESeq2. **(B)** Splenic ILCs, identified as lin$^-$ (CD3, B220, NK1.1) CD11b$^-$CD90.2$^+$CD127$^+$, from one-week-old *Clec9a*$^{cre/cre}$*Rosa*^{TOM} mice were analyzed for expression of CD11c, MHCII and RORγt. Plots are representative of three biological replicates. **(C)** TOM$^+$ cDC2 (red), RORγt$^-$TOM$^-$ DC2 (black) and RORγt$^+$TOM$^-$ DC2 (blue) from one-week-old *Clec9a*$^{cre/+}$*Rosa*^{TOM}*Rorc*^{eGFP} mice were identified and analyzed for surface expression of the indicated markers. **(D)** RORγt$^-$CD11b$^+$ DCs (black) and RORγt$^+$CD11b$^+$ DCs (blue) from one-week-old *Rorc*^{eGFP} mice were identified and compared for expression of indicated surface markers. **(C, D)** Data are representative of n=2.

Supplementary Figure 5

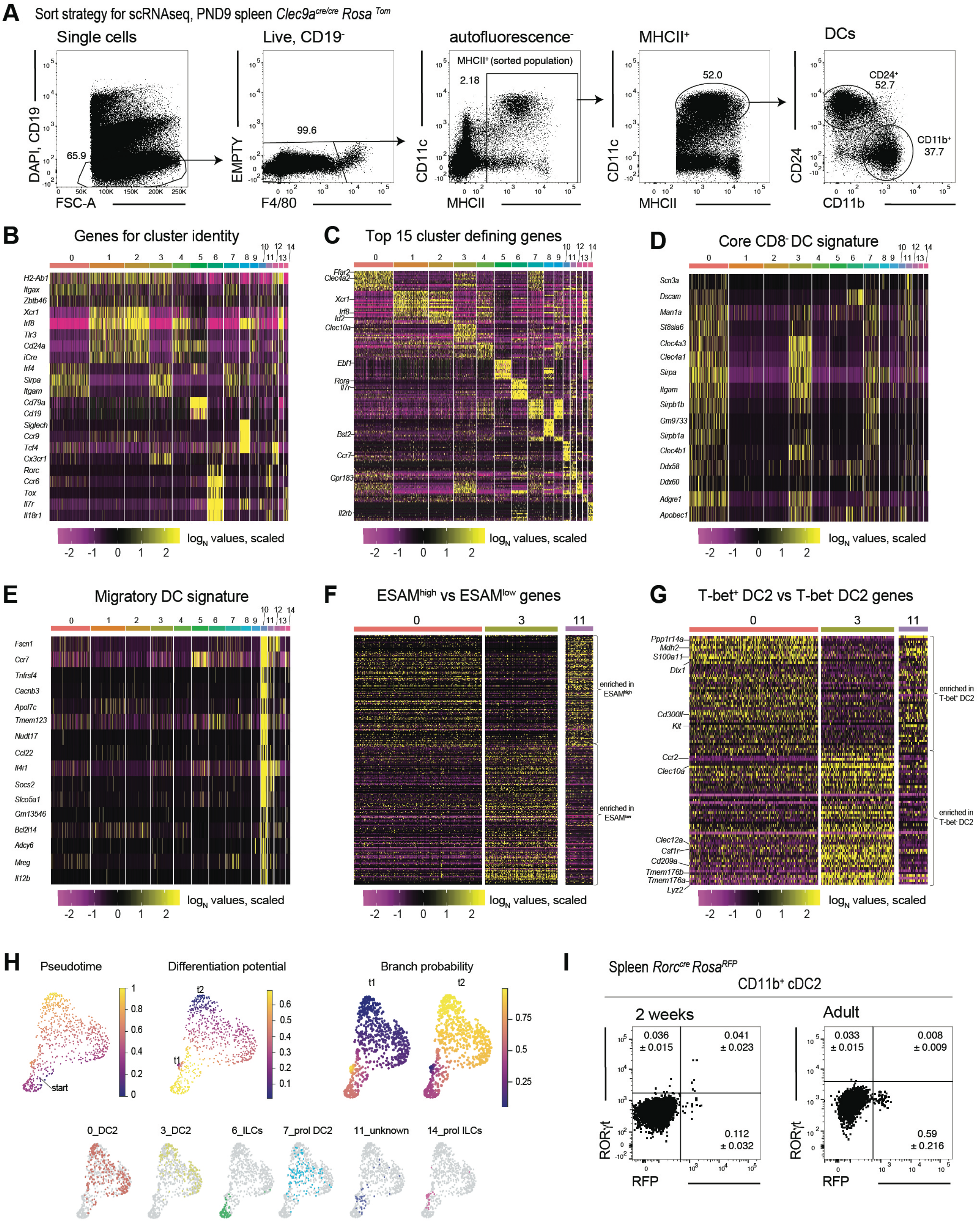

Supplementary Figure 5. TOM$^+$ cDC2 and TOM$^-$ DC2 in early life are transcriptionally identical. (A) Sort strategy for MHCII$^+$ cells from spleens of 9-day-old *Clec9a$^{cre/cre}$Rosa^{TOM}* mice for single cell RNA-seq. **(B)** Heatmap showing expression of genes used for cluster identification. **(C)** Heatmap displaying expression of the top 15 cluster defining genes. **(D-G)** Heatmaps displaying the expression of **(D)** core CD8$^-$ DC genes, **(E)** core migratory DC genes, **(F)** genes characteristic for ESAM^{high} and ESAM^{low} cDC2, as well as genes characteristic for T-bet$^+$ and T-bet$^-$ cDC2 **(G)** among cells of indicated clusters.**(B-G)** Expression is shown in logN values scaled by row. **(H)** Palantir pseudo-time analysis of differentiation potential and branch probabilities from cluster 11 (upper row). Start point and terminal states are indicated. The positioning of clusters 0, 3, 6, 7, 11 and 14 used for Palantir analysis is shown in tSNE embedding of the diffusion map (lower row). **(I)** Spleen from two-week-old and adult *Rorc^{cre}Rosa^{RFP}* mice were analyzed by flow cytometry. CD11c$^+$MHCII$^+$CD11b$^+$ cDC2 were identified and analyzed for the expression of RORγt and RFP. Numbers represent mean ± SD (n=7, two-week-old; n=4, adult). Source data are provided as a Source Data file.

Supplementary Figure 6

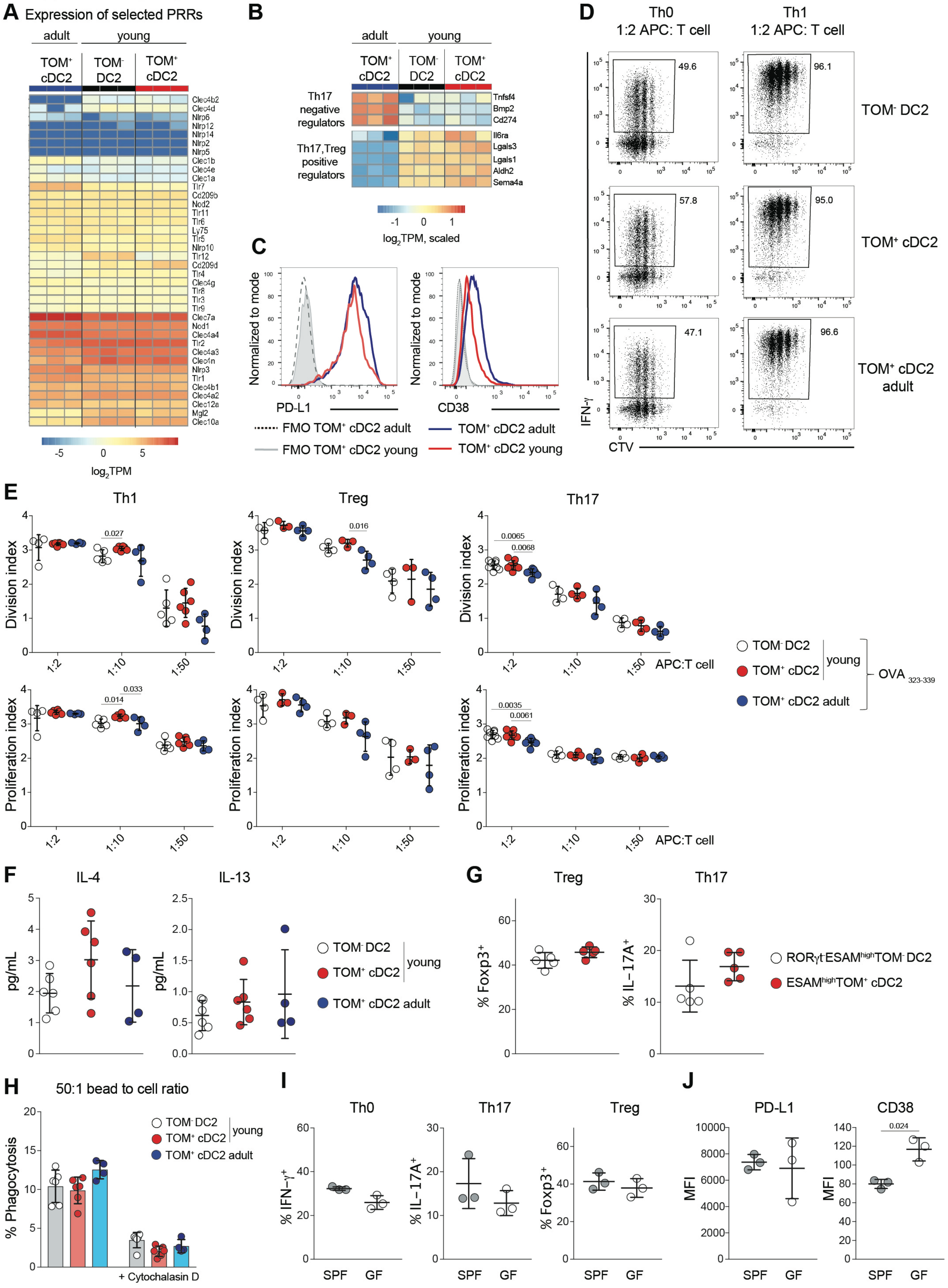

Supplementary Figure 6. TOM$^+$ cDC2 and TOM$^-$ DC2 can prime naïve T cell proliferation comparable to cDC2 from adults. (A, B) Sort-purified TOM$^-$ DC2 and TOM$^+$ cDC2 from 8-day-old and TOM$^+$ cDC2 from adult *Clec9a$^{cre/cre}$Rosa^{TOM}* mice were analyzed by mRNA sequencing. Heatmaps showing expression of selected pattern recognition receptors (log_2TPM values) **(A)** and genes involved in Th17 and Treg differentiation (log_2TPM values, scaled by row) **(B)**. **(C)** TOM$^+$ cDC2 from two-week-old and adult *Clec9a$^{cre/+}$Rosa^{TOM}* mice were analyzed by flow cytometry for PD-L1 and CD38 expression (n=4-7). **(D-F)** TOM$^-$ DC2 and TOM$^+$ cDC2 from two-week-old and TOM$^+$ cDC2 from adult *Clec9a$^{cre/+}$Rosa^{TOM}* mice were sorted, pulsed with $OVA_{323-339}$ and co-cultured with CTV-labelled OT-II cells as indicated. 3.5 days later OT-II cells were analyzed for cytokine expression **(D)** and cell division **(E)**. **(F)** Cytokine levels in supernatants of OT-II cells co-cultured with the indicated DC2 populations under Th0 conditions. Data in **(D-F)** are pooled from four independent experiments. **(G)** Sort-purified RORγt$^-$ESAM^{high}TOM$^-$ DC2 and ESAM^{high}TOM$^+$ cDC2 from two-week-old *Clec9a$^{cre/+}$Rosa^{TOM}Rorc-eGFP* mice were co-cultured with CTV-labelled OT-II cells in Treg or Th17 conditions for 3.5 days. The percentage of cytokine or Foxp3 positive proliferated OT-II cells is shown (n=5). **(H)** CD11c-enriched splenocytes from two-week-old (n=6) and adult (n=4) C*lec9a$^{cre/+}$Rosa^{TOM}* mice were cultured with yellow fluorescent beads at the indicated bead to cell ratio *in vitro* for 2 hours +/- cytochalasin D. Bead uptake was quantified by flow cytometry in TOM$^+$ cDC2 (Autofluoresence$^-$ CD11c$^+$MHCII$^+$CD11b$^+$TOMATO$^+$) and TOM$^-$ DC2 (Autofluoresence$^-$ CD11c$^+$MHCII$^+$CD11b$^+$TOMATO$^-$). **(I)** Sort-purified splenic CD11c$^+$MHCII$^+$CD11b$^+$ cDC2 from SPF or GF mice were co-cultured with CTV-labelled OT-II cells as indicated. 3.5 days later proliferated OTII cells were analyzed for cytokines and Foxp3 expression (n=3). **(J)** PD-L1 and CD38 expression on splenic cDC2 from SPF and GF mice (n=3). Each dot represents individual biological replicates **(E-H)** or mice **(I-J)**, horizontal bars represent mean, error bars represent SD. Comparison of TOM$^-$ DC2 and TOM$^+$ cDC2 was performed using two-tailed paired t test. Comparison of two-week-old and adult groups was performed using one-way ANOVA. Statistical analysis in **(I-J)** was performed using two-tailed t test. Only statistically significant comparisons are indicated. Source data are provided as a Source Data file.

Supplementary Figure 7

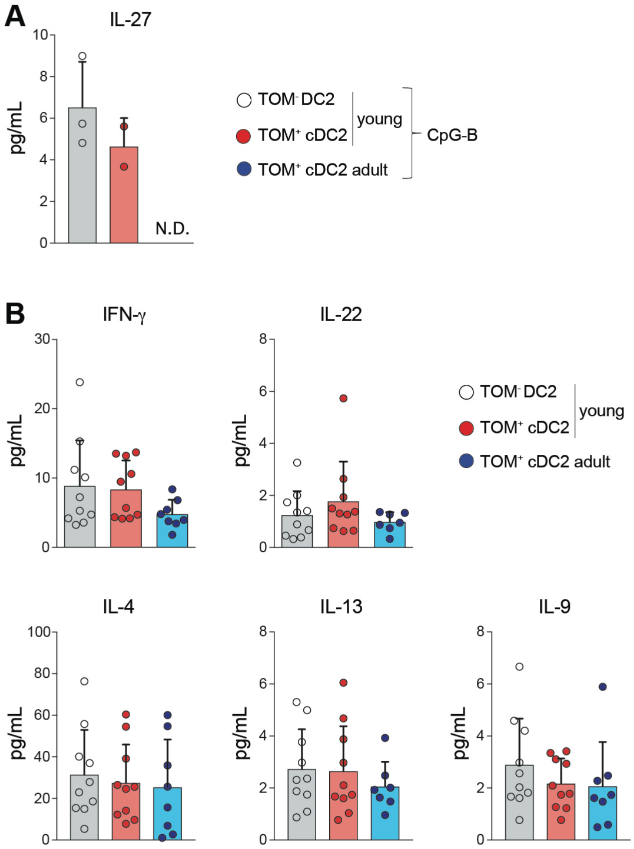

Supplementary Figure 7. Early life cDC2 show distinct responsiveness to CpG and induce distinct T cell responses upon targeted antigen delivery compared to adult life cDC2. (A) TOM^+ cDC2 and TOM^- DC2 from two-week-old and TOM^+ cDC2 from adult C*lec9a*$^{cre/+}$*Rosa*^{TOM} mice were sorted and stimulated with CpG-B. Cytokine production was analyzed 18-20h later (n=4, with IL-27 detected in 3 out of 4 replicates from two-week-old TOM^- DC2, 2 out of 4 replicates from two-week-old TOM^+ cDC2, while it was not detected in supernatants from adult TOM^+ cDC2). Each dot represents one biological replicate. **(B)** Two-week-old and adult *Clec9a*$^{cre/+}$*Rosa*^{TOM} mice were injected i.p. with anti-DCIR2-OVA antibody plus CpG-B. After 12 hours DC populations were sorted and co-cultured with OT-II T cells for 3.5 days. The indicated cytokines were quantified in supernatants from co-cultures (n=10, two-week-old; n=8, adult; IL-22 and IL-13 were detected in 7 out 8 adult replicates). Each dot represents one biological replicate from two independent experiments, horizontal bars represent mean, error bars represent SD. Statistical analysis was performed using two-tailed paired t test (comparing TOM^- DC2 and TOM^+ cDC2 groups) or one-way ANOVA (comparing two-week-old and adult groups). No statistically significant differences were found. Source data are provided as a Source Data file.
