# Supplementary material for: Environmental signals rather than layered ontogeny imprint the function of type 2 conventional dendritic cells in young and adult mice
Source: Nat Commun. 2021 Jan 19;12:464. doi: 10.1038/s41467-020-20659-2 (PMC7815729; doi:10.1038/s41467-020-20659-2)
Supplement: Supplementary file 3 — Reporting Summary [file 41467_2020_20659_MOESM3_ESM.pdf]

nature research

Corresponding author(s): Barbara U. Schraml

Last updated by author(s): Nov 25, 2020

Reporting Summary

Nature Research wishes to improve the reproducibility of the work that we publish. This form provides structure for consistency and transparency in reporting. For further information on Nature Research policies, see our Editorial Policies and the Editorial Policy Checklist.

Statistics

For all statistical analyses, confirm that the following items are present in the figure legend, table legend, main text, or Methods section.

n/a	Confirmed	
☐	☒	The exact sample size (*n*) for each experimental group/condition, given as a discrete number and unit of measurement
☐	☒	A statement on whether measurements were taken from distinct samples or whether the same sample was measured repeatedly
☐	☒	The statistical test(s) used AND whether they are one- or two-sided<br>*Only common tests should be described solely by name; describe more complex techniques in the Methods section.*
☒	☐	A description of all covariates tested
☐	☒	A description of any assumptions or corrections, such as tests of normality and adjustment for multiple comparisons
☐	☒	A full description of the statistical parameters including central tendency (e.g. means) or other basic estimates (e.g. regression coefficient) AND variation (e.g. standard deviation) or associated estimates of uncertainty (e.g. confidence intervals)
☐	☒	For null hypothesis testing, the test statistic (e.g. *F*, *t*, *r*) with confidence intervals, effect sizes, degrees of freedom and *P* value noted<br>*Give P values as exact values whenever suitable.*
☒	☐	For Bayesian analysis, information on the choice of priors and Markov chain Monte Carlo settings
☒	☐	For hierarchical and complex designs, identification of the appropriate level for tests and full reporting of outcomes
☒	☐	Estimates of effect sizes (e.g. Cohen's *d*, Pearson's *r*), indicating how they were calculated

Our web collection on statistics for biologists contains articles on many of the points above.

Software and code

Policy information about availability of computer code

Data collection	-Flow cytometry data was collected using BD FACSDiva Software (BD BioSciences, Version 8)<br>-Imaging data was collected using LAS X (Leica, Version 3.4.1.17670)
Data analysis	-FlowJo software (Tree Star, Inc.), version 10.6 was used for analysis of flow cytometry data.<br>-Microsoft Excel version 16.34 was used for table creation.<br>-GraphPad Prism 7 was used for graphical and statistical analysis.<br>-Fiji version 2.0.0 was used for analysis of imaging data.<br>-For bulk RNA sequencing, STAR (version 2.5.2a) was used to map RNA sequencing reads to the mouse genome (mm10). RSEM (version 1.2.31) was used to calculate expression of genes in transcript per million. RNA-seq analysis was performed in R (Version 3.5.3) with R-Studio (R-Studio Inc, Version 1.1.383). Differential gene expression analysis and principal component analysis was performed using DESeq2 (Version 1.22.2).Log2 fold change shrinkage was performed using the Apeglm package (Version 1.6.0). Heatmaps were generated using pheatmap (Version 1.0.12) and graphs were plotted with ggplot2 (Version 3.0.2).<br>-For single cell RNA sequencing, data was processed using the 10X Genomics Cell Ranger v3.0.2 pipeline. Sequencing reads were mapped to the mouse genome (mm10) using STAR (version 2.5.1b) after spiking in the sequences of iCRE (GenBank iD: AY056050.1), the predicted transcript of the unrecombined Rosa locus and Tomato sequence (GenBank ID: AY678269.1). Cell Ranger's count pipeline was run under default parameters. Further downstream analysis was conducted in R (Version 3.5.3) with R-Studio (R-Studio Inc, Version 1.1.383) using Seurat (v3.0.2) and SCtransform (version 0.3). For trajectory analysis the Palantir package was used.<br>-tSNE and UMAP algorithms (as part of DESeq2 and Seurat packages) were used for single cell and bulk RNA analyses.<br>-LEGENDplex™ cloud based program was used for analysis of cytokines in culture supernantants.

For manuscripts utilizing custom algorithms or software that are central to the research but not yet described in published literature, software must be made available to editors and reviewers. We strongly encourage code deposition in a community repository (e.g. GitHub). See the Nature Research guidelines for submitting code & software for further information.

Data

Policy information about availability of data

All manuscripts must include a data availability statement. This statement should provide the following information, where applicable:

- Accession codes, unique identifiers, or web links for publicly available datasets
- A list of figures that have associated raw data
- A description of any restrictions on data availability

The authors declare that the data supporting the findings of this study are available within the paper and its supplementary files. Source data are provided with this paper and raw data are available from the authors upon reasonable request. Datasets related to bulk and single cell sequencing experiments that were generated and analyzed for the current study have been deposited and made publicly available in the Gene Expression Omnibus under the accession number GSE151595 (https://www.ncbi.nlm.nih.gov/geo/query/acc.cgi?acc=GSE151595). The sequences of iCRE and Tomato used for the single cell RNA sequencing analysis are publicly available and can be found under the GenBank IDs AY056050.1 (https://www.ncbi.nlm.nih.gov/nuccore/AY056050) and AY678269.1 (https://www.ncbi.nlm.nih.gov/nuccore/AY678269), respectively.

Field-specific reporting

Please select the one below that is the best fit for your research. If you are not sure, read the appropriate sections before making your selection.

☒ Life sciences ☐ Behavioural & social sciences ☐ Ecological, evolutionary & environmental sciences

For a reference copy of the document with all sections, see nature.com/documents/nr-reporting-summary-flat.pdf

Life sciences study design

All studies must disclose on these points even when the disclosure is negative.

Sample size	No statistical methods were used to predetermine the sample size. All experiments were repeated at least 2 times to ensure an overall sample size of at least 3 per experimental group. The exact n values used to calculate the statistics are provided per experiment presented in the main and supplementary figures of the manuscript. For all experiments with neonatal and young mice, multiple mice were pooled in order to get sufficient number of cells.
Data exclusions	For single cell RNA sequencing analysis, cells with less than 200 genes detected or having more than 7.5% of mitochondrial associated genes were excluded from the analysis. Additionally, genes detected in less than 3 cells were removed from further analysis. These exclusion criteria are pre-established by the default parameters of the Seurat package and enable the removal of cells with poor sequencing quality (low number of total detected genes), dying cells (increased representation of mitochondrial associated genes) and of genes not reliably detected. For other experiments, when necessary data points were excluded if technical issues arose that prevented their reliable analysis (e.g. spread >30% between technical replicates in cytokine quantification of culture supernatants according to the manufacturer's recommendation).
Replication	Number of replicates is indicated in each experiments. In general, data presented are the result of at least 2 independent experiments with multiple biological replicates. All attempts for replication were successful.
Randomization	Mice were housed in the same room and rack in the respective animal facilities. Littermates were used for experiments whenever possible per developmental timepoint. For bulk and single cell RNA sequencing experiments mice were sex-matched.
Blinding	The investigators were not blinded during collection of animal tissues in experiments implementing mice of different age groups due to obvious differences in organ size. When mice of the same age but different genotypes were used, the investigators were blinded to their genotype during data collection and analysis. Genotyping was conducted by an independent person. In all experiments, samples were processed simultaneously or in parallel.

Reporting for specific materials, systems and methods

We require information from authors about some types of materials, experimental systems and methods used in many studies. Here, indicate whether each material, system or method listed is relevant to your study. If you are not sure if a list item applies to your research, read the appropriate section before selecting a response.

Materials & experimental systems

n/a	Involved in the study
	x Antibodies
x	Eukaryotic cell lines
x	Palaeontology and archaeology
	x Animals and other organisms
x	Human research participants
x	Clinical data
x	Dual use research of concern

Methods

n/a	Involved in the study
x	ChIP-seq
	x Flow cytometry
x	MRI-based neuroimaging

Antibodies

Antibodies used

All antibody details are listed in Supplementary Table 4 and also listed below:
Antibody used for flow cytometry-Clone-Source-Catalog number:
anti-CD3ε-Pacific Blue (PB) 145-2C11 Biolegend 100334
anti-CD3ε-Fluorescein isothiocyanate (FITC) 145-2C11 Biolegend 100306
anti-CD3ε-R-phycoerythrin-cyanine 5 (PE-Cy5) 145-2C11 Biolegend 100310
anti-CD4-PB RM4-5 Biolegend 100531
anti-CD4-PE-Cy5 RM4-5 Biolegend 100513
anti-CD4-PE-Cy7 RM4-5 Biolegend 100528
anti-CD4-FITC RM4-4 Biolegend 100406
anti-CD4-BUV737 GK1.5 BD 612844
anti-CD8a-PE-Cy5 53-6.7 Biolegend 100709
anti-CD8a-PB 53-6.7 Biolegend 100725
anti-CD8a-FITC 53-6.7 BD 553031
anti-CD11b-BV421 M1/70 Biolegend 101236
anti-CD11b-BV605 M1/70 Biolegend 101237
anti-CD11b-Allophycocyanin-cyanine 7 (APC-Cy7) M1/70 Biolegend 101226
anti-CD11b-BUV737 M1/70 BD 612800
anti-CD11c-BV785 N418 Biolegend 117336
anti-CD11c-Peridinin-chlorophyll (PerCP)-Cy5.5 N418 Biolegend 117328
anti-CD16/32 purified 2.4G2 BD 553142
anti-CD19-PB 6D5 Biolegend 115523
anti-CD19-BV650 6D5 Biolegend 115541
anti-CD19-FITC 6D5 Biolegend 115506
anti-CD24-FITC M1/69 Biolegend 101806
anti-CD24-BV605 M1/69 Biolegend 101827
anti-CD24-PE M1/69 Biolegend 101808
anti-CD24-BUV395 M1/69 BD 744471
anti-CD25-BV785 PC61 Biolegend 102051
anti-CD26-PE-Cy7 H194-112 Biolegend 137810
anti-CD26-APC H194-112 Biolegend 137807
anti-CD38-FITC 90 Biolegend 102705
anti-CD43-PE-Cy7 1B11 Biolegend 121218
anti-CD45.1-PB A20 Biolegend 110722
anti-CD45.1-FITC A20 Biolegend 110706
anti-CD45.2-PE-Cy7 104 Biolegend 109830
anti-CD45.2-PB 104 Biolegend 109820
anti-CD45R/B220-PB RA3-6B2 Biolegend 103227
anti-CD45R/B220-FITC RA3-6B2 Biolegend 103206
anti-CD45R/B220-PE RA3-6B2 Biolegend 103208
anti-CD45R/B220-PE-Cy5 RA3-6B2 Biolegend 103210
anti-CD45R/B220- Alexa Fluor (AF) 700 RA3-6B2 Biolegend 103232
anti-CD64-FITC X54-5/7.1 Biolegend 139316
anti-CD64-PE-Cy7 X54-5/7.1 Biolegend 139314
anti-CD64-APC X54-5/7.1 Biolegend 139306
anti-CD80-FITC 16-10A1 Biolegend 104706
anti-CD80-PE 16-10A1 Biolegend 104707
anti-CD86-BV605 GL-1 Biolegend 105037
anti-CD90.1-FITC OX-7 Biolegend 202504
anti-CD90.1-AF700 OX-7 Biolegend 202528
anti-CD90.1-PE-Cy7 OX-7 Biolegend 202517
anti-CD90.2-PE-Cy7 30-H12 Biolegend 105326
anti-CD90.2-AF700 30-H12 Biolegend 105320

anti-CD115-BV605 AFS98 Biolegend 135517
anti-CD117-PE-Cy7 2B8 Biolegend 105814
anti-CD127-BUV737 SB/199 BD 564399
anti-CD135-APC A2F10 Biolegend 135310
anti-CD135-PE A2F10 Biolegend 135306
anti-CD161c/NK1.1-PB PK136 Biolegend 108722
anti-CD161c/NK1.1-PE-Cy5 PK136 Biolegend 108716
anti-CD161c/NK1.1-FITC PK136 Biolegend 108706
anti-CD172a-PE-Cy7 P84 Biolegend 144008
nti-CD172a-BV605 P84 Biolegend 740390
anti-CD172a-PerCP-ef710 P84 Thermo Fisher Scientific 46-1721-82
anti-CD196/CCR6-BV421 29-2L17 Biolegend 129817
anti-CD274/PD-L1-BV421 10F.9G2 Biolegend 124315
anti-CD274/PD-L1-BV605 10F.9G2 Biolegend 124321
anti-CD370/DNGR-1-PE 1F6 Biolegend 92068
anti-F4/80-BV605 BM8 Biolegend 123133
anti-F4/80-BV785 BM8 Biolegend 123141
anti-F4/80-AF647 BM8 Biolegend 123122
anti-MHCIII A/I-E-AF700 M5/114.15.2 Biolegend 107622
anti-MHCII I-A/I-E-BV510 M5/114.15.2 Biolegend 107635
anti-MHCII I-A/I-E-PB M5/114.15.2 Biolegend 107620
anti-MHCII I-A/I-E-PerCP-ef710 M5/114.15.2 Thermo Fisher Scientific 46-5321-80
anti-Ly-6A/E-BV650 D7 Biolegend 108143
anti-Ly6C-PB HK1.4 Biolegend 128014
anti-Ly6C-FITC HK1.4 Biolegend 128005
anti-Ly6G-FITC 1A8 Biolegend 127606
anti-Ly6G-AF700 1A8 Biolegend 127616
anti-Ly6G-APC 1A8 Biolegend 127614
anti-SiglecH eFluor660 eBio440c Thermo Fisher Scientific 50-0333-82
anti-CLEC4A4/33D1-APC 33D1 Biolegend 124914
anti-XCR1-BV421 ZET Biolegend 148216
anti-XCR1-BV650 ZET Biolegend 148220
anti-V-alpha2 TCR-FITC B20.1 BD 553288
anti-V-alpha2 TCR-BUV395 B20.1 BD 743834
anti-TER-119-PB TER-119 Biolegend 116232
anti-TER-119-FITC TER-119 Biolegend 116206
anti-TER-119-PE-Cy5 TER-119 Biolegend 116209
anti-TER-119-BUV395 TER-119 BD 566206
anti-ESAM 1G8/ESAM Biolegend 136207
anti-Foxp3-AF647 150D Biolegend 320014
anti-RORγt-BV421 Q31-378 BD 562894
anti-IFN-γ-APC XMG1.2 Biolegend 505810
IFN-γ-PE/Dazzle 594 XMG1.2 Biolegend 505846
IFN-γ-BV650 XMG1.2 Biolegend 505831
anti-IL17A TC11-18H10.1 Biolegend 506907
anti-IL-4-PE 11B11 Biolegend 504104
anti-TNFa-PECy7 MP6-XT22 Biolegend 506324
anti-TNFa-PE MP6-XT22 Biolegend 506305

Antibody used for microscopy-Clone-Source-Catalog number:
anti-CD3ε-BV421 145-2C11 Biolegend 100336
anti-CD11b-AF647 M1/70 Biolegend 101220
anti-CD31-AF594 MEC13.3 Biolegend 102520
anti-MHCII A/I-E-AF488 M5/114.15.2 Biolegend 107616

Antibody used for in vivo targeting-Clone-Source-Catalog number:
αDCIR2-OVA 33D1 Dr. Diana Dudziak N/A

Validation

Commercially available antibodies have been validated by their respective vendors for species reactivity and application in flow cytometry and/or microscopy. Validation data are available on the manufacturer's website using the catalog number of each product. All commercial antibodies were further tested by flow cytometry in our lab on murine splenocytes or bone marrow cells to define their dilution factor, by performing serial dilution experiments.
Antibodies used for in vivo targeting experiments have been validated in previous studies PMIDs: 17204652 and 28389502

Animals and other organisms

Policy information about studies involving animals; ARRIVE guidelines recommended for reporting animal research

Laboratory animals	Throughout this study, mice of various ages were used as indicated in the respective experiments. Mice over the age of 8 weeks were considered adults. The neonatal period was defined as the first 10 days of life. Mice younger than 3 weeks of age were euthanized by decapitation. Adult mice were euthanized by cervical dislocation. Mice were sex-matched but male and female mice were used for all experiments. In most experiments, littermates were used. When littermates could not be used, e.g. in experiments involving mice of different age, all mice were kept under the same barrier conditions and in the same racks. Only germ-free mice and the respective SPF controls used were not co-housed/bred under the same barrier.<br>Mice were maintained in specific pathogen-free SPF conditions with a 12 hour dark/light cycle, in individually vented cages (IVCs, type II long cages, measuring 18x30x13 cm with stocking density according to EU guideline 2010/63) supplied with autoclaved bedding, play tunnels, nestles and mouse houses. Irradiated food and sterile filtered and UV-light exposed water were provided ad libitum. Cage manipulations took place in laminar flow hoods. Air temperature was 22 ± 2°C and humidity 55 ± 10% with daily control and record.<br><br>The following mouse strains were bred at the Biomedical Center or Walter-Brendel- Centre for Experimental Medicine in specific pathogen-free conditions:<br>-Clec9atm2.1(icre)Crs (Clec9a-cre) (Jackson Laboratory Stock No: 025523)<br>-Gt(ROSA)26Sortm1(EYFP)Cos (Rosa26lox-STOP-lox-EYFP) (Jackson Laboratory Stock No: 006148)<br>-Gt(ROSA)26Sortm9(CAG-tdTomato)Hze (Rosa26lox-STOP-lox-tdtomato) (Jackson Laboratory Stock No: 007909)<br>-Tg(Csf1r-cre/Esr1*)1Jwp (Csf1rMer-iCre-Mer) (Jackson Laboratory Stock No: 019098)<br>-Flt3ltm1Imx (Flt3l−/−) (MMRRC Stock No: 37395-JAX)<br>-Mybtm1Ssp (Myb+/-) (Jackson Laboratory Stock No: 004757)<br>-Rag1tm1(cre)Thr (Rag1cre)<br>-Tg(TcraTcrb)425Cbn (OT-II) (Jackson Laboratory Stock No: 004194) crossed to a Thy1.1 (CD90.1) background<br>-Tg(Rorc-EGFP)1Ebe (RorceGFP)<br>-Cxcr4creER<br>-Gt(ROSA)26Sortm4(ACTB-tdTomato,-EGFP)Luo/J (RosamTmG) (Jackson Laboratory Stock No: 007576)<br>-Ly5.1 (CD45.1) B6.SJL (Jackson Laboratory Stock No: 002014)<br>-C57BL/6J<br><br>The following mouse strains were bred at the Institute of Immunology and Immunotherapy, College of Medical and Dental Sciences, University of Birmingham, Birmingham:<br>-Tg(Rorc-cre)1Litt (Rorc-cre) (Jackson Laboratory Stock No: 022791)<br>-l7rtm1.1(icre)Hrr (IL7Rcre)<br>-Gt(ROSA)26Sortm1Hjf (Rosa26lox-STOP-lox-RFP) (EMMA strain ID EM:02112)<br><br>The following mouse strains were bred at the University Hospital Erlangen, Germany:<br>-Ifnar1tm1Agt (Ifnar-/-) (MMRRC Stock No: 32045-JAX)<br><br>Germ Free mice were provided by Dirk Haller.
Wild animals	No wild animals were used in this study.
Field-collected samples	No field-collected samples were used in this study.
Ethics oversight	All animal procedures were performed in accordance with national and institutional guidelines for animal welfare and approved by the Regierung of Oberbayern.

Note that full information on the approval of the study protocol must also be provided in the manuscript.

Flow Cytometry

Plots

Confirm that:

- [ ] The axis labels state the marker and fluorochrome used (e.g. CD4-FITC).
- [x] The axis scales are clearly visible. Include numbers along axes only for bottom left plot of group (a 'group' is an analysis of identical markers).
- [ ] All plots are contour plots with outliers or pseudocolor plots.
- [x] A numerical value for number of cells or percentage (with statistics) is provided.

Methodology

Sample preparation	Cell isolation for flow cytometry:<br>Spleens were minced into small pieces and digested in 1mL of RPMI with 200U/mL collagenase IV and 0.2mg/mL DNAse I for 30 min at 37°C while shaking. After digestion, cells were passed through a 70µm strainer and washed once with FACS buffer

(PBS, 1% fetal calf serum (FCS), 2.5mM EDTA, 0.02% sodium azide). Erythrocytes were lysed with Red Blood Cell Lysing Buffer Hybri-Max for 2min at room temperature, washed once and resuspended in FACS buffer for further analysis. Bone marrow from adult mice was isolated from femurs and tibiae by flushing, bone marrow from mice under two weeks of age was isolated by crushing the bones through a 70µm cell strainer. Erythrocytes were lysed as above, and cells were resuspended in FACS buffer for further analysis. Liver was minced into small pieces and digested in 2mL PBS containing Mg2+ and Ca2+ with 1mg/mL collagenase IV, 60U/mL DNAse I, 2.4mg/mL Dispase II and 3% FCS for 30min at 37°C while shaking. After digestion, cells were passed through a 100µm strainer and centrifuged for 3min at 50g at 4°C in order to pellet hepatocytes. The supernatant was collected and recentrifuged for 7min at 320g at 4°C. Pelleted cells were resuspended in FACS buffer for further analysis.

Cell isolation for cell sorting and functional analyses:
Cell isolation from spleen was performed as above but FACS buffer without sodium azide was used for all functional and RNA profiling experiments. For OT-II cell isolation spleen was mechanically disrupted through a 70µm strainer and washed once with FACS buffer without sodium azide. For targeting experiments, splenocytes were isolated by mechanically disrupting the spleens through a 70µm strainer and washing once with PBS containing 1% FCS.

Instrument

LSR Fortessa (BD Biosciences), Aria III Fusion (BD Biosciences)

Software

Data Collection: BD FACSDiva Software (BD BioSciences), version 8.
Data Analysis: FlowJo software (Tree Star, Inc.), version 10.6.

Cell population abundance

Sample purity was confirmed post sort by flow cytometry and cells counts were quantified using CountBright™ Absolute Counting Beads (Thermo Fisher Scientific).

Gating strategy

All samples were initially gated on FSC-A vs SSC-A plot, douplet exclusion was performed using FSC-A vs FSC-H and FSC-A vs SSCW plots and live cells were gated selecting cell negative for DAPI or fixable viability dye eFluor™ 780. Autofluorescent red pulp macrophages were identified by plotting F4/80 vs an empty channel. Cell populations were identified as follows and gating strategies are shown in Fig 1, Suppl Fig 1, Suppl Fig 2, Suppl Fig 3 and Suppl Fig 5:
DCs: autofluorescence-CD11c+MHCII+CD64-(CD24+XCR1+/- or CD11b+ESAMhigh/low), Bone marrow derived DCs form FLT3L cultures: CD45.2+B220-SiglecH-CD11c+MHCII+(CD24+ or CD172a+), Bone marrow pre-cDCs: lin-MHCII-CD11blowCD11c+ CD135+CD172aint, Splenic pre-cDCs: lin-MHCII-CD11blowCD11c+CD43+CD135+CD172aint, CDPs: lin-CD11c-MHCII-CD11blowCD115+CD135+CD117low, MDPs: lin-CD11c-MHCII-CD11blowCD115+CD135+CD117high, CLPs: lin-CD11b-/lowCD115-CD117intSca-1intCD135+CD127+, Bone marrow TER119+ cells: lin-CD117-TER119+, Splenic macrophages: F4/80highCD11blow, Neutrophils: Ly6G+CD11bhigh or Ly6C+CD11bhigh, B cells: CD19+ or CD11c-B220+, Splenic ILCs: lin-CD11b-CD90.2+CD127+, pDCs: CD11cintB220+, OT-II T cells after co-culture with APCs: CD90.1+CD4+TCRVa2+CD11c-

☒ Tick this box to confirm that a figure exemplifying the gating strategy is provided in the Supplementary Information.
