# Supplementary material for: Environmental signals rather than layered ontogeny imprint the function of type 2 conventional dendritic cells in young and adult mice
Source: Nat Commun. 2021 Jan 19;12:464. doi: 10.1038/s41467-020-20659-2 (PMC7815729; doi:10.1038/s41467-020-20659-2)
Supplement: Supplementary file 4 — Description of Additional Supplementary Files [file 41467_2020_20659_MOESM4_ESM.pdf]

Description of Additional Supplementary Files

Supplementary Data 1: Differentially expressed genes comparing TOM+ cD
C2 and TOM- DC2 from 8-day-old mice.

Supplementary Data 2: Differentially expressed genes comparing TOM+ and TOM- cells from clusters 0,3,11 of single cell RNA-Sequencing data.

Supplementary Data 3: Differentially expressed genes comparing TOM+ cD
C2 from 8-day-old mice and TOM+ cDC2 from adult mice.

Supplementary Data 4: List of antibodies used in this study
